# Incorporating immune cell surrogates into a full-thickness tissue equivalent of human skin to characterize dendritic cell activation

**DOI:** 10.1038/s41598-024-81014-9

**Published:** 2024-12-04

**Authors:** Johanna Maria Hölken, Anna-Lena Wurz, Katja Friedrich, Patricia Böttcher, Dounia Asskali, Holger Stark, Jörg Breitkreutz, Timo Buhl, Lars Vierkotten, Karsten Rüdiger Mewes, Nicole Teusch

**Affiliations:** 1 Institute of Pharmaceutical Biology and Biotechnology, Heinrich Heine University Düsseldorf, Universitätsstr.1, 40225 Düsseldorf, Germany; 2 Henkel AG & Co. KGaA, Henkelstr. 67, 40589 Düsseldorf, Germany; 3 Institute of Pharmaceutical and Medicinal Chemistry, Heinrich Heine University Düsseldorf, Universitätsstr.1, 40225 Düsseldorf, Germany; 4 Institute of Pharmaceutics and Biopharmaceutics, Heinrich Heine University Düsseldorf, Universitätsstr.1, 40225 Düsseldorf, Germany; 5 Department of Dermatology, Venereology and Allergology, University Medical Center Göttingen, Robert-Koch-Straße 40, 37099 Göttingen, Germany

**Keywords:** Full-thickness skin model, Skin equivalent, Langerhans cells, Dermal dendritic cells, CD1a, CD207, Cell biology, Immunology

## Abstract

In the past decades studies investigating the dendritic cell (DC) activation have been conducted almost exclusively in animal models. However, due to species-specific differences in the DC subsets, there is an urgent need for alternative in vitro models allowing the investigation of Langerhans cell (LC) and dermal dendritic cell (DDC) activation in human tissue. We have engineered a full-thickness (FT) human skin tissue equivalent with incorporated LC surrogates derived from the human myeloid leukemia-derived cell line Mutz-3, and DDC surrogates generated from the human leukemia monocytic cell line THP-1. Topical treatment of the skin models encompassing Mutz-LCs only with nickel sulfate (NiSO_4_) or 1-chloro-2,4-dinitrobenzene (DNCB) for 24 h resulted in significant higher numbers of CD1a positive cells in the dermal compartment, suggesting a sensitizer-induced migration of LCs. Remarkably, exposure of the skin models encompassing both, LC and DDC surrogates, revealed an early sensitizer-induced response reflected by increased numbers of CD1a positive cells in the epidermis and dermis after 8 h of treatment. Our human skin tissue equivalent encompassing incorporated LC and DDC surrogates allows the investigation of DC activation, subsequent sensitizer identification and drug discovery according to the principles of 3R.

**Supplementary Information:**

The online version contains supplementary material available at 10.1038/s41598-024-81014-9.

## Introduction

The key players of the cutaneous immune response include epidermal and dermal dendritic cells^1^. Epidermal dendritic cells, known as Langerhans cells (LCs), are located predominantly suprabasal in the stratum spinosum with extended cell protrusions reaching into the stratum corneum^2,3^. Dermal dendritic cells (DDCs) are located throughout the dermis, but mostly underneath the epidermal-dermal junction^4^. In fact, due to their epidermal location, Langerhans cells were considered for a long time to function as the exclusive key regulators of the cutaneous immune response. However, contact hypersensitivity studies (CHS) in LC-deficient mouse models led to the concept of three different outcomes, varying from a diminished sensitization^5^, to an enhanced sensitization^6^ and an unchanged response, proposing LCs to be dispensable for CHS^7^. Furthermore, a central role for dermal dendritic cells in contact hypersensitivity was suggested as dermal dendritic cells were found to colonize in distinct areas of the lymph node. Studies indicate that DDCs might migrate faster and significantly outnumber LCs in lymph nodes^7^. However, these studies were exclusively conducted in mice and therefore limited in terms of prediction. Noteworthy, an overlap of only ~ 30% of skin associated genes was identified comparing the mouse and the human genome^8^.

The genetic discrepancy as well as fundamental differences in skin anatomy and in cutaneous immune cell populations, particularly in dendritic cell subsets, might explain translation failures from murine models to the human system^9^. As species-specific differences lead to controversial discussions regarding the precise roles and molecular events of both dermal dendritic cell types in human skin immunity and contact hypersensitivity, there is an urgent need for alternative *in vitro* models allowing the investigation of the human immune response according to the criteria of the 3R principles (“replace”, “reduce”, “refine”). In the past years, skin equivalents containing Langerhans cell surrogates derived from CD14^+^ peripheral blood mononuclear cells (PBMCs)^10,11^, from cord-blood-derived CD34^+^ hematopoietic progenitor cells^12,13^ or from the human myeloid leukemia-derived cell line Mutz-3^11,14–16^ have been reported. Accordingly, in our recent study we were able to generate a human immune competent full-thickness (FT) skin model by incorporating functional dermal dendritic cell surrogates derived from the monocytic cell line THP-1 allowing the qualitative identification of potential sensitizers or drug candidates^17^. However, to date, only one human FT skin model including LC as well as DDC surrogates, both derived from CD14^+^ PBMCs, has been described for analyzing the impact of ultraviolet (UV) stress on cutaneous immune cells^10^. According to our knowledge, no human FT skin model comprising functional LC and DDC surrogates aiming at exploring skin sensitization or inflammation has been reported yet although both DC cell types are considered to be crucial mediators regulating skin immunity and homeostasis.

Upon cutaneous infectious, inflammatory or sensitizing stimuli, such as nickel sulfate (NiSO_4_) or 1-chloro-2,4-dinitrobenzene (DNCB), LCs as well as DDCs capture and phagocytose the hapten-protein complex, undergo a maturation process, which is accompanied by the induction of several molecular pathways, and migrate to skin draining lymph nodes to subsequently activate T cells^1,18^. LCs are mainly characterized by a distinctively high expression of Cluster of Differentiation (CD)1a and CD207 (also named Langerin)^9,20^. While the function of CD1a and CD207 surface marker expression on LCs still remains elusive, several studies indicate antigen presenting roles for both surface markers^21–26^. CD207/Langerin is not only expressed on the cell surface, but has been identified as the main molecular component of Birbeck granules, which are formed as subdomains of endosomal recycling compartments upon langerin accumulation^27^, suggesting the role in antigen uptake and degradation. So far, the potential to recognize, uptake and degrade viral particles, glycoproteins and glycolipid antigens has been described^21–23^. Furthermore, a CD1a mediated antigen presentation of lipid antigens to T- cells, promoting skin inflammation, was shown in various studies^24–26^. In steady state conditions, it is presumed that the adhesion of Langerhans cells to keratinocytes is mediated by E-cadherin^28^. Upon LC differentiation and maturation, the E-cadherin expression is proposed to become downregulated^29,30^ and the expression of C-X-C chemokine receptor type 4 (CXCR4) is induced for a stromal cell-derived factor 1 (SDF-1) (secreted by fibroblasts) mediated migration to the dermis^31,32^. In LCs and DDCs, phagocytosis of hapten-protein complexes is accompanied by the upregulation of the major histocompatibility complex (MHC) class II, such as the Human Leukocyte Antigen–DR isotype (HLA-DR), required for the presentation of antigens to CD4^+^ T cells^33^. Furthermore, high expressions of the maturation markers Cluster of Differentiation (CD)80 and CD86 are induced and essential for the co-stimulation of naïve CD4^+^ T cells via their CD28 and cytotoxic T-lymphocyte-associated protein 4 (CTLA-4)/(CD152) receptors^34^. In addition, high expression of CD83 stimulates the CD86 surface marker expression and stabilizes the MHC II surface expression on activated DCs, thereby promoting the stimulation, proliferation, and maturation of naïve CD4^+^ T cells^35,36^.

In summary, we have engineered a human immune competent full-thickness skin model encompassing incorporated and functional DDC surrogates, described earlier as THP-1-derived CD14^-^, CD11c^+^ immature dendritic cells (iDCs)^17,37^ on one hand, and containing CD1a^+^, CD207^+^ LC surrogates derived from the human myeloid leukemia-derived cell line Mutz-3 on the other hand, allowing the molecular characterization of human DC activation in vitro upon compound treatment according to the 3R criteria.

## Materials and methods

### Langerhans cell (LC) surrogates

Mutz-3 cells (DSMZ, #ACC 295, Braunschweig, Germany) were maintained at a cell density of 2 × 10^5^ cells/mL in 12 well plates in MEM α (Gibco, #12561056, Grand Island, NY, USA) supplemented with 20% FBS (Gibco, #22400089), 1% P/S (Gibco, #15140122), 0.05 mM 2-mercaptoethanol (Gibco, #21985023) and 200 U/mL rhGM CSF (ImmunoTools, #11343125, Friesoythe, Germany).

For the generation of LC surrogates 2 × 10^5^ Mutz-3 cells/mL were seeded in 2 mL MEM α (Gibco, #12561056) supplemented with 5% FBS (Gibco, #22400089), 1% P/S (Gibco, #15140122) and 0.05 mM 2-mercaptoethanol (Gibco, #21985023) into a 12-well plate. For differentiation, the following cytokines were added: 1000 U/mL rhGM-CSF (ImmunoTools, #11343125), 400 U/mL TGF-β (ImmunoTools, #11343160) and 100 U/mL TNF-α (PeproTech, #300-01A, Rocky Hill, NJ, USA). The cells were incubated for 9 days at 37 °C, 5% CO_2_ without medium exchange.

### Immature dendritic cell (iDC) surrogates

iDC were generated according to our previous protocol^37^. Briefly 2 × 10^5^ THP-1 cells/mL were differentiated with 1500 U/mL rhGM-CSF (ImmunoTools, #11343125) and 1500 U/ml IL-4 (ImmunoTools, #11340045) over 5 days with medium exchange on day 3.

### Incorporation of Mutz-LCs and iDCs into full-thickness skin models

Feeder cells (Phenion, #hFeeder, Henkel AG & Co. KGaA, Düsseldorf, Germany) were seeded with 2.5 × 10^5^ in 11 mL keratinocyte medium (Phenion, #K CM-250, Henkel AG & Co. KGaA) into a T75 flask. After 3 days 2.5 × 10^5^ primary human foreskin keratinocytes from juvenile donors (Phenion, #hK P1, Henkel AG & Co. KGaA) were seeded onto the feeder cells. After 6 days of cultivation, feeder cells were detached by incubation with 0.05% trypsin (Gibco, #25300054) for 2 min at 37 °C, 5% CO_2_ and keratinocytes were harvested using 0.05% trypsin for 6 min, 37 °C, 5% CO_2_. For Mutz-LC models, 2.5 × 10^5^ keratinocytes in P2 were seeded together with 1 × 10^6^ Mutz-LCs in 1 mL keratinocyte medium (Phenion, #K CM-250, Henkel AG & Co. KGaA) onto dermis models based on a solid and porous collagen matrix and primary human foreskin fibroblasts (kindly provided by Henkel AG & Co. KGaA, Düsseldorf, Germany). For Mutz LC + iDC models, 2.5 × 10^5^ keratinocytes in P2 were seeded together with 5 × 10^5^ Mutz-LCs and 5 × 10^5^ THP-1 derived iDCs in 1 mL keratinocyte medium onto the dermis models. After 2 h of incubation at 37 °C, 5% CO_2_, 2.5 × 10^5^ freshly detached keratinocytes in were seeded in 1 mL keratinocyte medium on top of the Mutz-LC/Mutz-LC + iDC models. After 24 h of incubation at 37 ° C, 5% CO_2_ the medium was exchanged. After further 24 h submerse phase, the skin models were lifted into the Air-liquid Interface (ALI) and cultivated with Air-liquid Interface Culture Medium (Phenion, #ALI CM HC-250, w/o hydrocortisone, Henkel AG & Co. KGaA) for 10 days. For treatment, sensitizers (380 µM NiSO_4_ and 20 µM DNCB) or the respective solvent control (0.2% DMSO in PBS) were applied topically, by carefully pipetting 30 µl of the test substances onto the skin models.

### Surface marker detection via flow cytometry

Cells were harvested after differentiation or treatment and washed in autoMacs Running Buffer (Miltenyi Biotec, #130-091-221, Gladbach, Germany). At least 1 × 10^5^ cells for each antibody panel were transferred to 96-well u-bottom plates and incubated in Automacs Running Buffer with the following antibodies (1:50): REA Control (S)-VioGreen (Miltenyi Biotec, #130-113-444), REA Control (S)-PE (Miltenyi Biotec, #130-113-438), REA Control (S)-APC (Miltenyi Biotec, #130-113-434); REA Control (S)-PE-Vio770, (Miltenyi Biotec, #130-113-440); HLA-DR-VioGreen (Miltenyi Biotec, #130-111-948), CD1a-PE (Miltenyi Biotec, #130-112-022); CD207-PE-Vio770 (Miltenyi Biotec, #130-112-370), CD54-APC (Miltenyi Biotec, #130-121-342); CD86-APC (Miltenyi Biotec, #130-116-161), CD83-PE (Miltenyi Biotec, #130-110-561), CD11b-VioGreen (Miltenyi Biotec, #130-110-617), CD11c-APC (Miltenyi Biotec, #130-113-584) for 10 min in the dark. The cells were washed twice with autoMacs Running Buffer. To determine the cell viability, cells were stained with DAPI (10 µg/mL) (Sigma-Aldrich, #D9542, Darmstadt, Germany). Flow cytometry analysis was performed using the CytoFlex (B5-R3-V5) from Beckman Coulter (Brea, CA, USA).

### Western blot analysis

2 × 10^5^ Mutz-LCs/mL were seeded in 4 mL MEM α supplemented with 5% FBS, 1% P/S and 0.05 mM 2-mercaptoethanol into a 12-well plate and treated with DNCB [25 µM] (Sigma-Aldrich, #237329) or NiSO_4_ [500 µM] (Sigma-Aldrich, #227676) for 30 min or 1 h, respectively. Sample preparation, determination of protein concentrations, SDS-Page and western blotting was performed as published previously^17^. The following primary antibodies were used: phospho-p38 MAPK (Thr180/Tyr182) (Cell Signaling Technology, #4511T, Danvers, MA, USA), p38 MAPK (Cell Signaling Technology, #8690T), IκBα (Cell Signaling Technology, #9242S) and vinculin (Cell Signaling Technology, #13901S). Secondary antibody incubation was performed using the respective horseradish peroxidase-coupled secondary antibody (Goat anti-Rabbit (H + L), Thermo Fisher Scientific, #31460, Waltham, MA, USA). Antibody binding was detected with the SuperSignal West Pico Plus substrate kit (Thermo Fisher Scientific, #34577) in a ChemStudio Imager (Analytik Jena, #849-97-0928-04, Jena, Germany).

### Skin model dissociation and RNA isolation

For qPCR analysis epidermis and dermis were separated by incubation in thermolysin (0.5 mg/mL) (#T7902, Sigma-Aldrich) for 2 h at 4 °C. The separated epidermis and dermis were minced into small pieces. RNA isolation was performed using the RNeasy Mini Kit (Qiagen, #74104, Düsseldorf, Germany), the DNase Kit (Qiagen, #79254) and proteinase K (Qiagen, #19133). Enzymatic dissociation was achieved by incubation with the RLT buffer from the RNeasy Kit at 20 °C for 45 min followed by an incubation step with proteinase K for 30 min at 55 °C, 400 RPM. After centrifugation, the supernatant was mixed with 0.7x of the volume of 98% ethanol and spinned through a RNeasy spin column. One washing step with RW1 buffer was performed before applying RNase/RDD solution (10 µl + 70 µl) from the DNase Kit for at least 15 min. The following steps were performed according to the manufacturer’s instructions of the RNeasy Mini Kit.

### Real-time quantitative PCR (RT-qPCR)

Mutz-LCs were seeded as described for Western blot analysis and treated with DNCB [20 µM] (Sigma-Aldrich, #237329) or NiSO_4_ [380 µM] (Sigma-Aldrich, #227676) for 6 h. RNA extraction, cDNA synthesis and qPCR was performed as published in former studies^17,37^. The specific primers used are listed in Table 1. After amplification, a threshold was set for each gene and Ct values were calculated for all samples.

**Table 1:** Primer sequences used for RT-qPCR

	Forward (5′ → 3′)	Reverse (5′ → 3′)
GAPDH	TGCACCACCAACTGCTTAGC	GGCATGGACTGTGGTCATGAG
IL-6	GGCACTGGCAGAAAACAACC	GCAAGTCTCCTCATTGAATCC
IL-8	ACTGAGAGTGATTGAGAGTGGAC	AACCCTCTGCACCCAGTTTTC
IL-1α	TGTATGTGACTGCCCAAGATGAAG	AGAGGAGGTTGGTCTCACTACC
IL-1β	GCACGATGCACCTGTACGAT	CACCAAGCTTTTTTGCTGTGAGT
IL-12p40	TGTCGTAGAATTGGATTGGTATC	AACCT GCCTCCTTTGTG
TNF-α	CCCTGCTGCACTTTGGAGTG	TCGGGGTTCGAGAAGATGAT
CD1a	CGCACCATTCGGTCATTTGAGG	TCCTGAGACCTTTCCAGAGTGC
CD207	TAATCTGCCTGACGCTGGTCCT	GGTGCTGATGTTGTCCACACGA
CD86	CCATCAGCTTGTCTGTTTCATTCC	GCTGTAATCCAAGGAATGTGGTC
CD83	TCCTGAGCTGCGCCTACAG	GCAGGGCAAGTCCACATCTT
CXCR4	CTCCTCTTTGTCATCACGCTTCC	GGATGAGGACACTGCTGTAGAG
CCR7	CAACATCACCAGTAGCACCTGTG	TGCGGAACTTGACGCCGATGAA
E-cad	CGAGAGCTACACGTTCACGG	GGGTGTCGAGGGAAAAATAGG

### Cryosectioning and immunofluorescence staining

Cryosectioning and immunofluorescence staining were performed as described previously^17^. Briefly, skin models were cryosectioned into 7 µm slices and blocked in 10% goat serum (Invitrogen, #50062Z, Waltham, MA, USA). Primary antibody incubation was conducted using: Cytokeratin 5 (OriGene, DM361, Rockville, MD, USA) (1:75), CD1a (Santa Cruz Biotechnology, #sc-18885, Dallas, TX, USA) (1:50) and CD45-VioBright R667 (Miltenyi Biotec, #130-110-779) (1:50). Secondary antibody staining was performed using Alexa Flour 488 (Invitrogen, #A11017) (1:200) and Alexa Flour 546 (Invitrogen, #A11018) (1:200) combined with DAPI staining (10 µg/ml) (Sigma, ##D9542).

### Haematoxylin and eosin staining

Haematoxylin and eosin staining’s were performed according to our previous protocol^17^. Briefly, skin models were fixed in formaldehyde, dehydrated, paraffin embedded and cut into 5 µm sections. The hematoxylin and eosin staining was conducted using an automated procedure (Thermo Scientific, #Gemini AS). Imaging was performed using an Olympus microscope (BX51, Camera Olympus DP7).

### Whole slide imaging and quantification

Imaging of the whole skin tissue slices was performed using a confocal spinning disc imaging system (CQ1, Yokogawa, Ratingen, Germany). To obtain focused images, the 40x objective was chosen and a region with at least four sections and a total of 70-80 field of views (FOVs) (≙ 4 − 6 sections with 6–10 × 2 FOVs) were defined for each slide depending on the length and alignment of the tissue slice. The whole slide image quantification of CD1a positive cells was carried out with the help of the analysis software CellPathfinder, High Content Analysis Software (Version 3.06.02.06) (URL: https://www.yokogawa.com/library/documents-downloads/software/lsc-cellpathfinder-software/) (Yokogawa). First, the single FOVs were aligned and threshold values (grey level) were defined to identify the epidermal compartment and the integrated LC surrogates. In a final step the range of the size filter for LCs was set to >10.7 µm to exclude cell debris, representing putative false positive counts.

### Statistical evaluation

Statistical evaluation was performed using GraphPad Prism version 8.4.3 (GraphPad Software, Inc., San Diego, CA, USA). Statistical significances were calculated using an unpaired t-test, one-way ANOVA with Dunnett’s multiple comparisons test or two-way ANOVA with Sidak’s multiple comparisons test. The significance levels were defined and referred to as *p ≤ 0.05; **p ≤ 0.01; ***p ≤ 0.001; ****p ≤ 0.0001.

## Results

Langerhans cells are primarily distinguished from other dendritic cell subtypes by their pronounced CD207/Langerin and CD1a expression^19,20^.

**Fig. 1:**
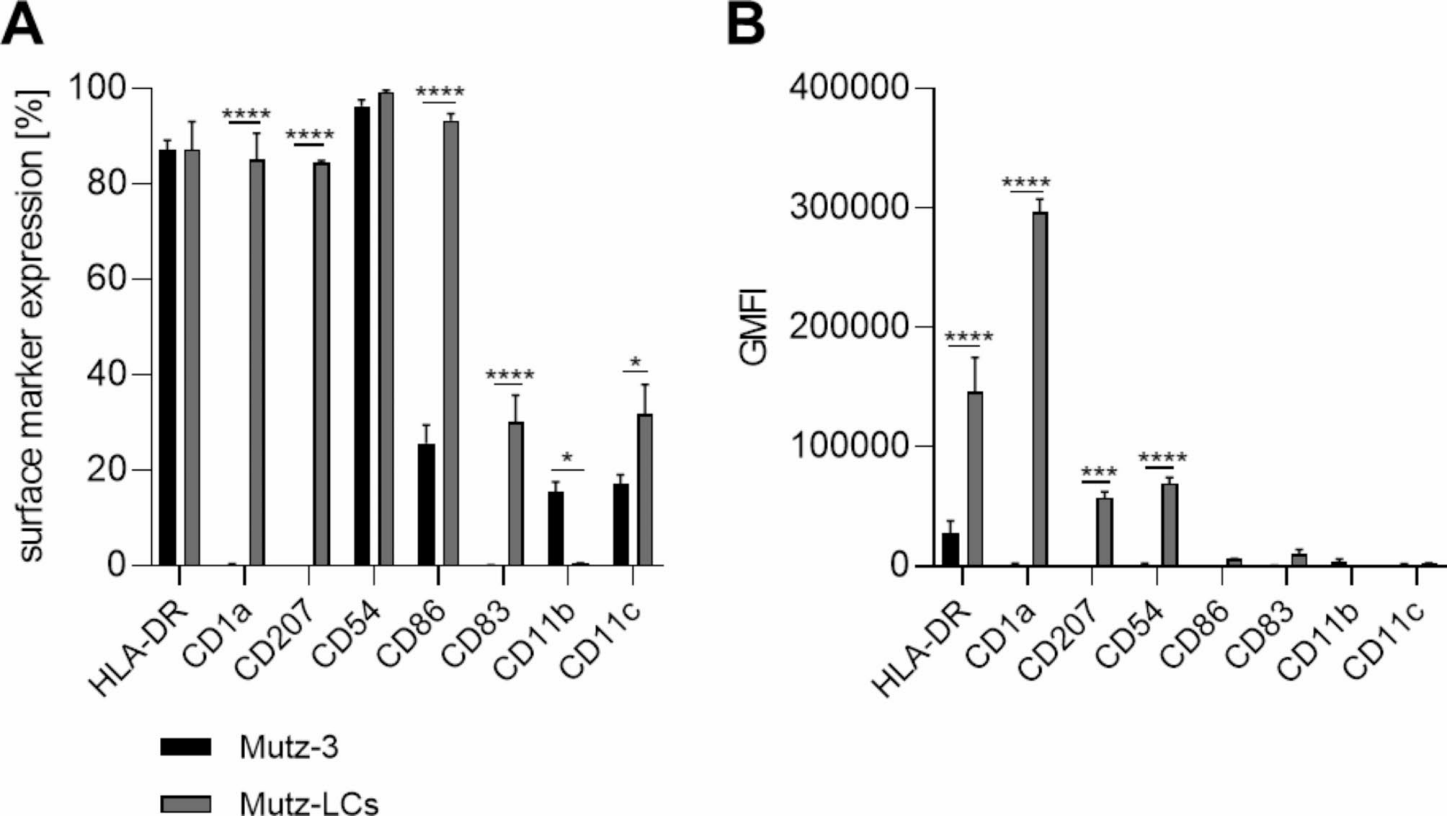
Surface marker expression of undifferentiated Mutz-3 cells and Mutz-LCs. (**A**) Surface marker expression depicted as percentage of positive cells (**B**) Surface marker expression depicted as geometric mean fluorescence intensity (GMFI). Mutz-3 cells were differentiated into Mutz-LCs with 1000 U/mL GM-CSF, 400 U/mL TGF-β and 100 U/mL TNF-α for 9 days. Surface marker expression of at least 10,000 viable cells was analyzed via flow cytometry. Error bars indicate the standard errors of the mean (n = 3 independent experiments with *p ≤ 0.05, ***p ≤ 0.001, and ****p ≤ 0.0001).

The successful differentiation of Mutz-3 cells into LC surrogates in the presence of GM-CSF, TGF-β and TNF-α was proven by the induction of CD1a (~ 85%) and CD207 (~ 84.5%) surface marker expression compared to control. In addition, the expression of CD86 (~ 3.6-fold), CD83 (~ 158-fold) and CD11c (~ 1.9-fold) could also be significantly increased (Fig. 1A). Moreover, the geometric mean fluorescence intensity (GMFI), depicting the brightness and relative measure of antigen abundance, was significantly enhanced for HLA-DR (~ 5.2-fold), CD1a (~ 221-fold), CD207 (157~ fold) and CD54 (~ 41-fold) (Fig. 1B). In line with previously published marker expression patterns for LC surrogates after treatment with skin sensitizers^38^, we were able to induce a significant increase in the number of CD83 positive cells (~ 3.0-fold) and a significant increase of the GMFI for HLA-DR (~ 3.2-fold) after NiSO_4_ treatment [380 µM] for 24 h. However, DNCB [20 µM] treatment led only to minor increase in the number of CD83 positive cells (~ 1.5-fold) and a minor increase of the GMFI for HLA-DR (~ 1.3-fold). In contrast, compared to the solvent control, the GMFI for CD1a was significantly decreased 24 h after exposure to NiSO_4_ [380 µM] (~ 1.8-fold) or DNCB [20 µM] (~ 2.0-fold) (Supplementary Fig. 1).

**Fig. 2:**
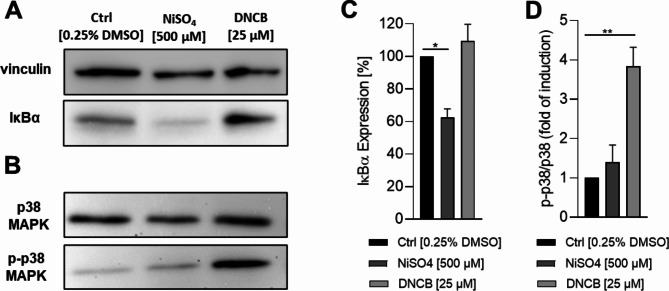
Degradation of IκBα after NiSO_4_ [500 µM] and DNCB [25 µM] treatment for 1 h (**A** and **C**) and phosphorylation of p38 MAPK after NiSO_4_ [500 µM] and DNCB [25 µM] treatment for 30 min (**B** and **D**). (**A**) and (**B**) depict one representative blot of three independent experiments. (**C**) and (**D**) Quantification of image bands normalized to the solvent control. Error bars indicate the standard errors of the mean (n = 3 independent experiments with *p ≤ 0.05 and **p ≤ 0.01).

Alongside the mentioned changes in the LC-specific and DC-specific activation and maturation markers, it is known for DCs that sensitization and activation is accompanied by sensitizer-specific activation of distinct inflammatory pathways such as the nuclear factor (NF)-κB and the p38 mitogen activated protein kinase (MAPK) pathways^17,39–41^. Moreover, loss of function studies revealed, that the activation of the NF-κB pathway via IκBα degradation upon treatment with nickel salts and the phosphorylation of p 38 MAPK upon DNCB exposure is crucial for the upregulation of CD80, CD86 and CD83 and therefore fundamentally involved in the maturation of DCs^39,41^. However, this was proven for various DC surrogates, including DDCs^17,38–41^, but not for Mutz-LCs yet. To confirm the activation of the signalling pathways mentioned, Mutz-LCs were treated with either NiSO_4_ [500 µM] for 1 h or DNCB [25 µM] for 30 min. Compared to the control, treatment of Mutz-LCs with NiSO_4_, but not with DNCB, resulted in a significant degradation of IκBα (~ 1.6-fold) (Fig. 2A and C). In contrast, while treatment with DNCB led to a significant phosphorylation of p38 MAPK (~ 3.8-fold) in Mutz-LCs, treatment with NiSO_4_ resulted only in a minor phosphorylation induction of p38 MAPK (~ 1.4-fold) (Fig. 2B and D). Hence, Mutz-3 derived LC surrogates reveal a similar sensitizer-specific induced activation pattern of intracellular inflammatory pathways, namely NF-κB and p38 MAPK, as published for DCs, including DDC surrogates^17,38–41^, suggesting a fundamental role of both pathways in the maturation of LCs as well.

**Fig. 3:**
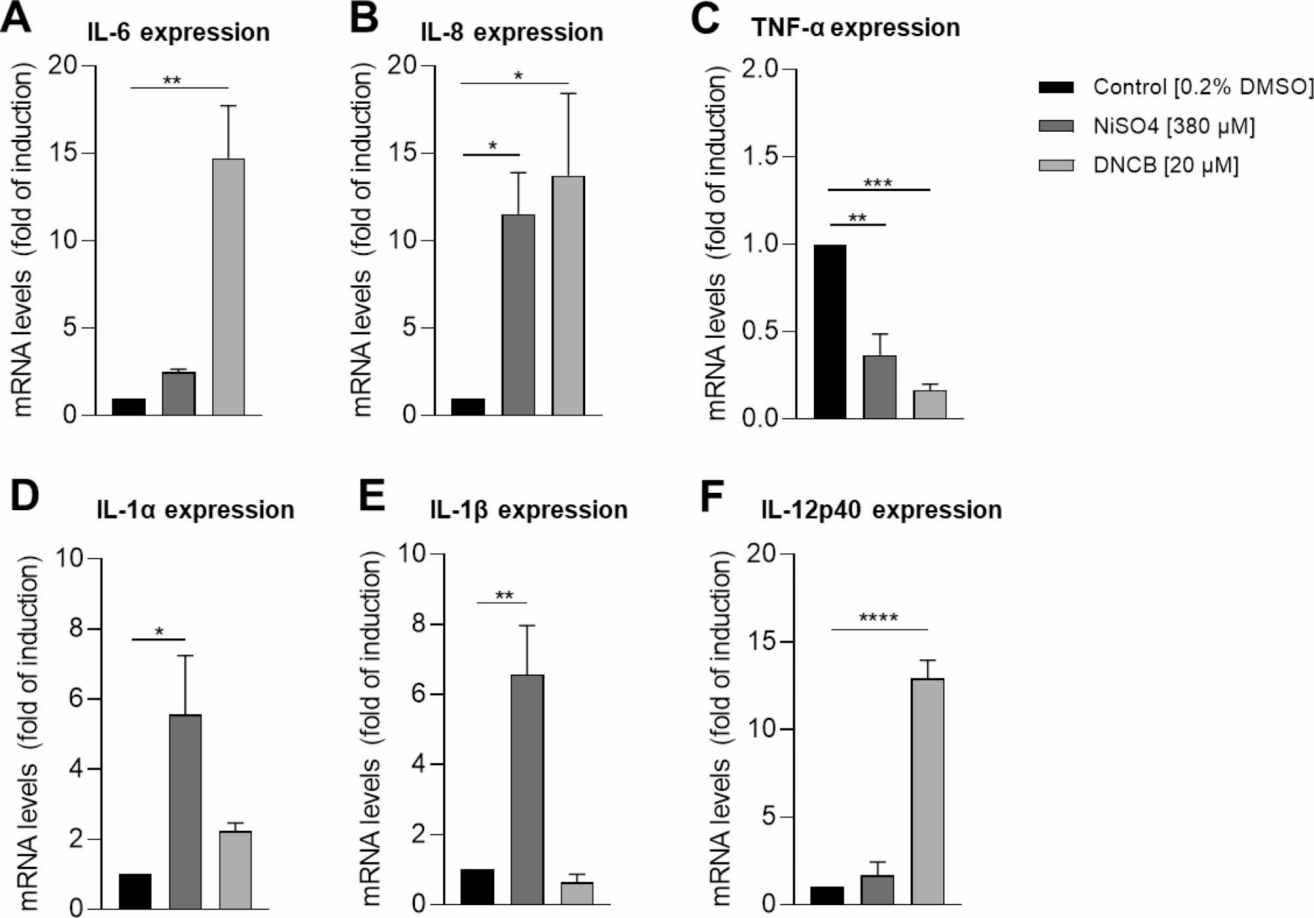
mRNA levels of inflammatory cytokine expression by Mutz-LCs: (**A**) IL-6, (**B**) IL-8, (**C**) TNF-α (**D**) IL-1α, (**E**) IL-1β and (**F**) IL-12p40, after NiSO_4_ [380 µM] and DNCB treatment [25 µM] for 6 h. Results are depicted as fold of induction compared to the solvent control [0.2% DMSO] and normalized to the expression of the housekeeping gene [GAPDH]. Error bars indicate the standard errors of the mean (n = 3 independent experiments with *p ≤ 0.05, **p ≤ 0.01, ***p ≤ 0.001, and ****p ≤ 0.0001).

Furthermore, loss of function studies for the NF-κB and the p38 MAPK pathway in PBMC as well as in cord-blood derived DCs, revealed the central role of both pathways in the secretion of inflammatory cytokines such as interleukin (IL)-1, IL-8 or IL-12p40^39–41^, required for T cell activation in cutaneous CHS^42–44^. In line, former studies confirmed the sensitizer induced upregulation of IL-8, IL-6 and IL-12p40 in dermal dendritic cell surrogates^17,37^. To prove whether Mutz-LCs are capable to upregulate inflammatory cytokines after sensitizer treatment, mRNA levels of IL-6, IL-8, TNF-α, IL-1α and IL-1β and IL-12p40 were analyzed (Fig. 3). Treatment of Mutz-LCs with NiSO_4_ [380 µM] resulted in significant higher mRNA levels of IL-8 (~ 11.5 fold), IL-1α (~ 5.6-fold) and IL-1-β (~ 6.6-fold), but only in a minor induction for IL-6 (~ 2.5-fold). Treatment with DNCB [25 µM] revealed significant higher mRNA levels of IL-6 (~ 14.7-fold) and IL-8 (~ 13.7-fold), but only a minor induction of IL-1α (~ 2.2-fold) and no significant change for IL-1β mRNA levels. Furthermore, both DNCB and NiSO_4_ treatment of Mutz-LCs lowered mRNA levels for TNF-α (NiSO_4_: 2.7-fold; DNCB: ~ 6-fold). Comparable to dermal dendritic cell surrogates^37^, Mutz-LCs treated with DNCB upregulated mRNA levels of IL-12p40 significantly, while treatment with NiSO_4_ led only to a minor induction of IL-12p40 (NiSO_4_: ~ 1.7-fold; DNCB: ~ 13-fold) (Fig. 3). Overall, depending on the applied sensitizer, Mutz-LCs might be able to secrete inflammatory cytokines essential for the activation and recruitment of T cells in the skin.

**Fig. 4:**
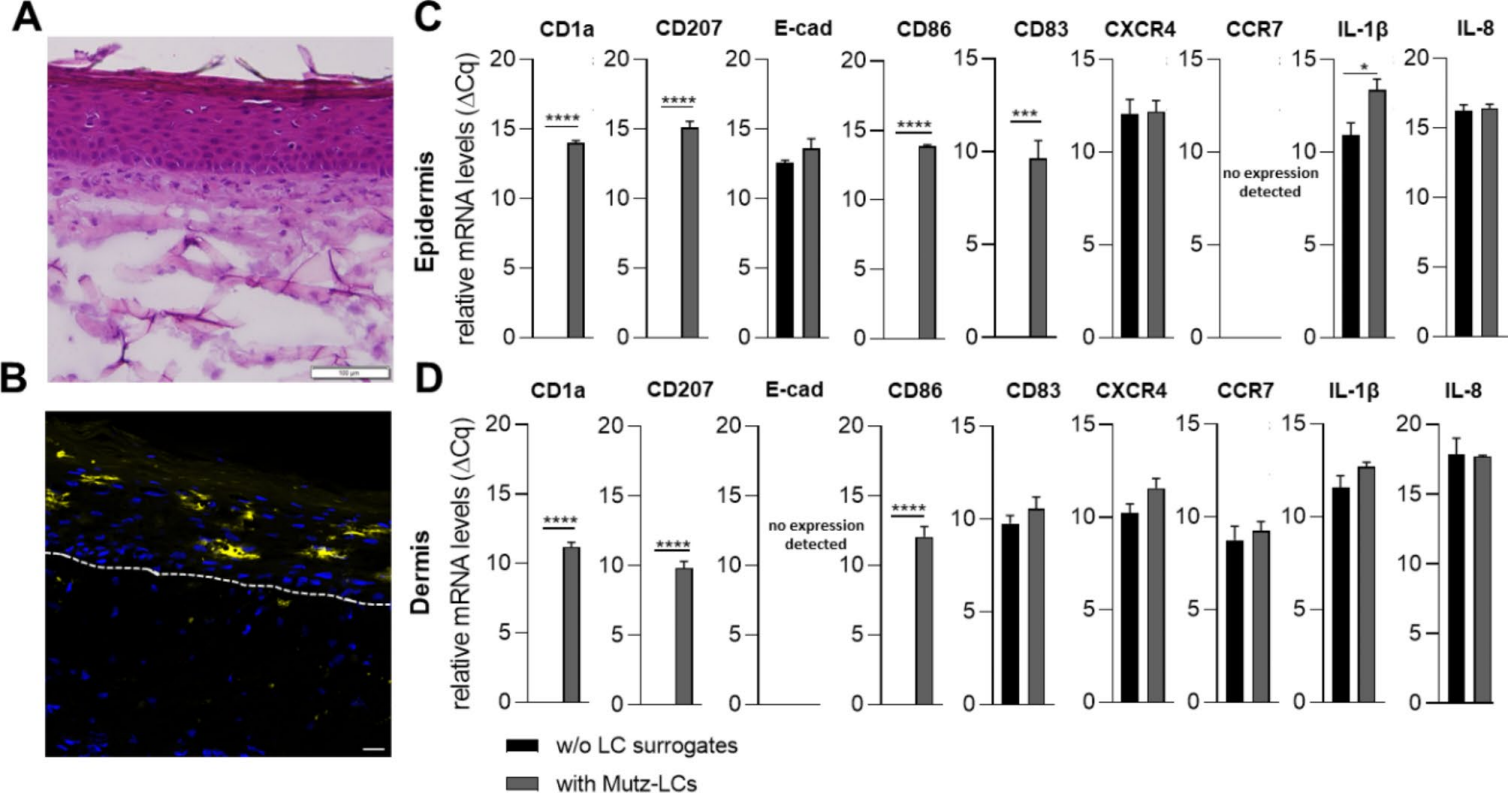
Integration of Mutz-LCs into a full-thickness skin model. (A) H&E staining of the full-thickness skin model including LC surrogates. Scale Bar = 100 µm. (**B**): Immunofluorescent staining skin model including LC surrogates. LC surrogates were stained with CD1a (yellow signal) and nuclei were stained with DAPI (Blue signal). Scale bar = 20 µm. (**C-D**) Analysis of the relative mRNA levels (ΔCq) of LC markers, maturation and migration markers and cytokines expressed by the epidermal (**C**) and dermal (**D**) compartment in the regular full-thickness skin model vs. the full-thickness skin model with incorporated LC surrogates. Epidermis and dermis of the full-thickness model without and with incorporated LC surrogates were separated and dissociated enzymatically and RNA was extracted for cDNA synthesis for RT-qPCR. Error bars indicate the standard errors of the mean (n=3 independent experiments with *p ≤ 0.05, ***p ≤ 0.001 and ****p ≤ 0.0001)

After confirming the ability of isolated Mutz-LCs to demonstrate a molecular Langerhans cell response in the presence of common sensitizers such as NiSO_4_ and DNCB in 2D, we integrated the Mutz-LCs into the engineered skin equivalent. In line with published data^11,14,15^, we were able to integrate Mutz-3 derived LC surrogates into the epidermis of the skin model. Integration of Mutz-LCs did not impair the epidermis (Fig. 4A). The integrated Mutz-LCs were identified and visualized via immunofluorescence staining with CD1a (Fig. 4B). When compared to the regular skin models, integration of Mutz-LCs resulted in a significant upregulation of mRNA levels for CD1a, CD207, CD86, CD83 and IL-1β in the epidermis. mRNA levels of E-cadherin, IL-8 and CXCR4 were only slightly increased after the integration of Mutz-LCs. CCR7 mRNA levels could not be detected in the epidermis, even after incorporating Mutz-LCs (Fig. 4C). Similar to the epidermis, integration of Mutz-LCs significantly increased mRNA levels of CD1a, CD207 and CD86 in the dermis, but there was no influence on the mRNA levels of CD83, CXCR4, CCR7, IL-1β and IL-8 (Fig. 4D). Hence, integration of Mutz-LC surrogates did not change the epidermal differentiation. Most importantly, the LC integration did not lead to an increase of the pro-inflammatory interleukin-8, related to the severity of skin inflammation^45^ and therefore often used as a biomarker for sensitizer identification^46,47^.

**Fig. 5:**
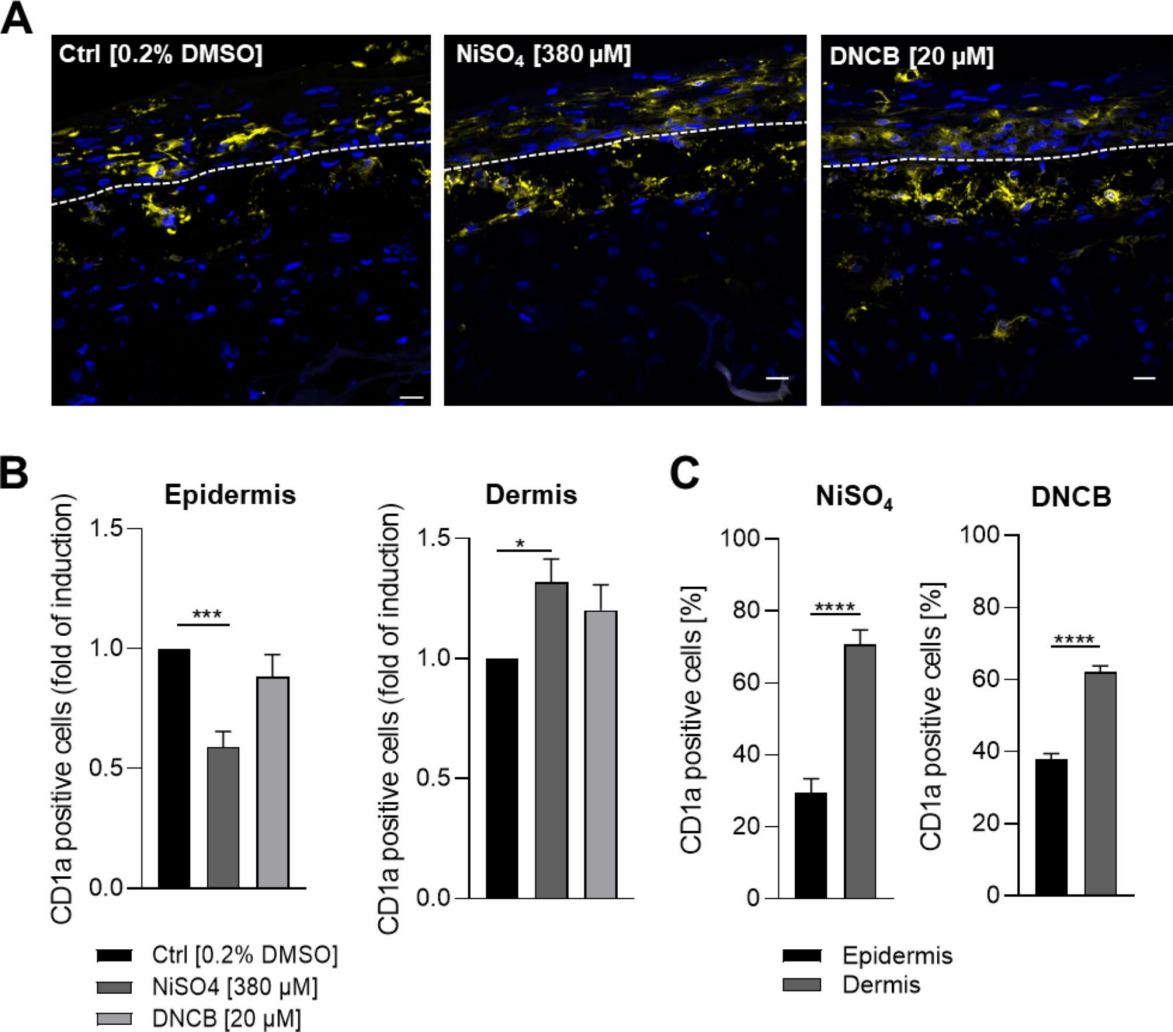
Histological analysis of the full thickness skin model with incorporated LC surrogates. Skin models were topically treated with NiSO_4_ [380 µM] and DNCB [20 µM] for 24 h. (**A**) Immunofluorescent staining of the full-thickness skin model tissue including LC surrogates after treatment with solvent control or sensitizers. LC surrogates were stained with CD1a (yellow signal). Nuclei were stained with DAPI (blue signal). Scale bar = 20 µm. Sensitizer induced migration of the LC surrogates from the epidermis to the dermal compartment was quantified via whole slide image analysis and depicted as fold of induction for CD1a positive cells in the epidermis and dermis compared to the solvent control (**B**) and as distribution in percentage (**C**). Error bard indicate the standard errors of the mean (n=3 independent experiments with *p ≤ 0.05, **p ≤ 0.01 and ***p ≤ 0.001)

To prove the functionality and immune competence of the LC surrogates in the tissue equivalent, the skin models were treated topically with either NiSO_4_ [380 µM], DNCB [20 µM] or the solvent control DMSO for 24 h. In line with reported studies, but to our knowledge the first to quantify whole slide images, we were able to prove significant lower numbers of CD1a positive cells in the epidermis (~ 1.7-fold) and a pronounced higher number of CD1a positive cells in the dermal compartment (~ 1.3-fold) after NiSO_4_ treatment compared to the solvent control (Fig. 5A,B). Furthermore, a comparable trend of a lower number of CD1a positive cells in the epidermis (~ 1.1-fold) and higher numbers of CD1a positive cells in the dermis (~ 1.2-fold) was found after DNCB treatment (Fig. 5A,B). Notably, whole slide image quantification of the CD1a signal revealed significant higher numbers of CD1a positive cells in the dermis compared to the respective epidermal compartment after NiSO_4_ (~ 2.4-fold) as well as after DNCB (~ 1.6-fold) treatment (Fig. 5C), suggesting a sensitizer-induced migration of CD1a positive LC-surrogates.

**Fig. 6:**
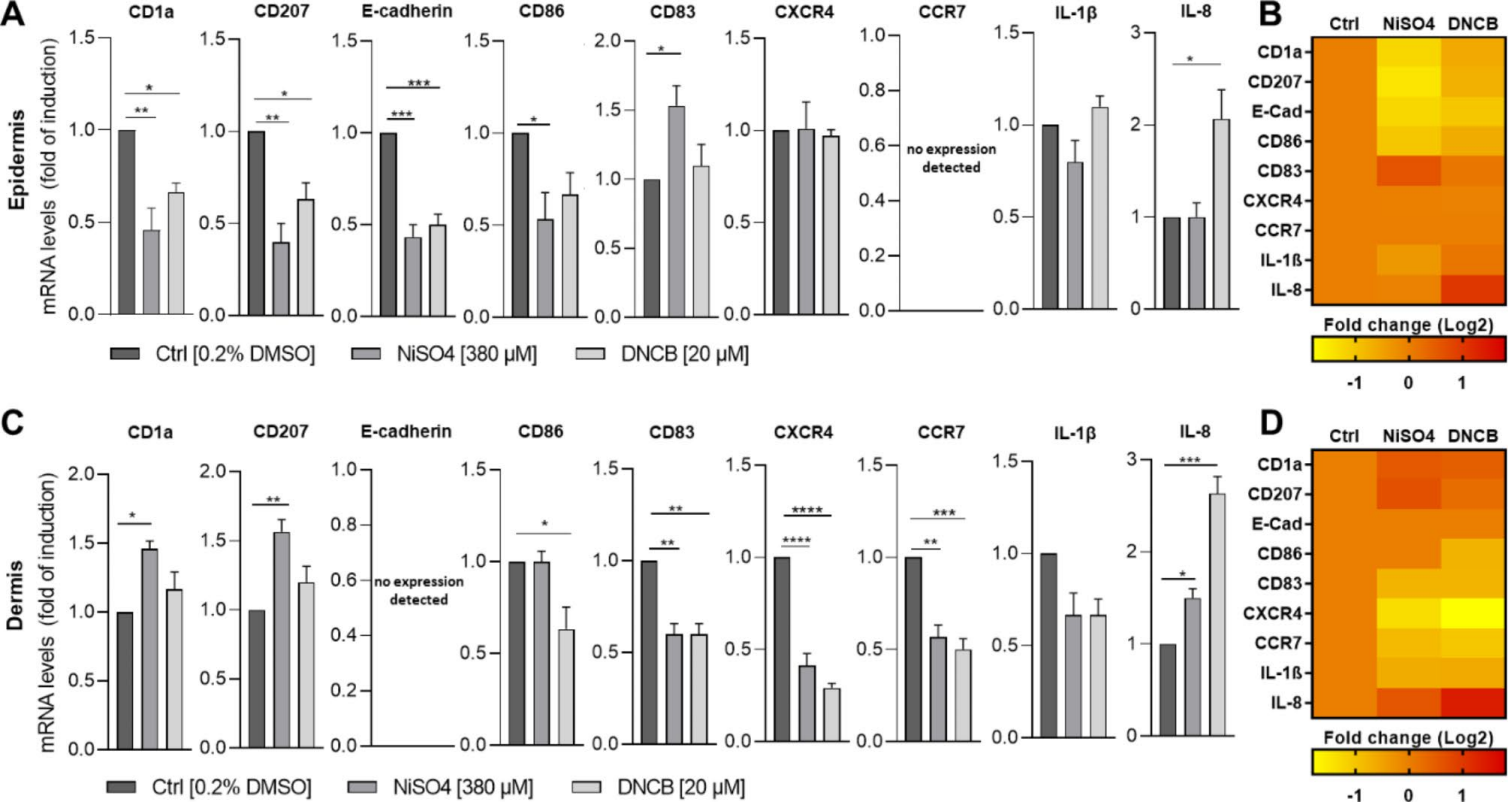
Analysis of the mRNA levels of LC markers, maturation and migration markers and cytokines expressed by the epidermal (**A-B**) and dermal (**C-D**) compartment in the regular full-thickness skin model vs. the full-thickness skin model with incorporated LC surrogates after topical application of sensitizers. 24 h after application of NiSO_4_ [380 µM] or DNCB [20 µM] the regular full-thickness skin models and the full-thickness skin models with incorporated LC surrogates were separated into epidermis and dermis and mechanically and enzymatically dissociated. RNA was extracted for cDNA synthesis for RT-qPCR. (**A** and **B**) Results are depicted as fold of induction/fold change compared to the solvent control [0.2% DMSO] and normalized to the expression of the housekeeping gene [GAPDH]. (C & D) Heatmap of the RT-qPCR analysis as fold change (Log2). Error bard indicate the standard errors of the mean (n=3 independent experiments with *p ≤ 0.05, ***p ≤ 0.001 and ****p ≤ 0.0001).

So far, the presumed molecular events of LC activation, maturation and migration were mostly concluded from *in vivo* experiments in mice, which do not represent the cutaneous anatomy and immune cell population of human skin^9^. To investigate these molecular events in the engineered skin tissue equivalent after incorporating LC surrogates, we analyzed the mRNA levels in the epidermal versus the dermal compartment of LC specific markers (CD1a, CD07), of maturation markers (CD83, CD86), of markers assumed to be involved in migration (E-cadherin, CXCR4 and CCR7) and of inflammatory cytokines (IL-1β, IL-8) 24 h after topical treatment with NiSO_4_ [380 µM] or DNCB [20 µM]. Results of the ΔΔCt values are indicated as fold of induction compared to the solvent control and normalized to the housekeeping gene (GAPDH) (Fig. 6A,C). The proportional changes for each marker analyzed, was calculated as the Log2 fold change, outlining the upregulation (+1) and downregulation (− 1) of the mRNA levels for each specific marker compared to the solvent control, and illustrated in the form of a heatmap (Fig. 6B,D). First of all, topical DNCB treatment of the skin model with incorporated LC surrogates led to significant lower CD1a (~ 1.5-fold) and CD207 (~ 1.6-fold) mRNA levels in the epidermis and 1.2-fold induced mRNA levels in the dermis for CD1a and CD207 compared to the solvent control. Similarly, treatment with NiSO_4_ resulted in a significant decrease of mRNA levels for CD1a (~ 2.2-fold) and CD207 (~ 2.5-fold) in the epidermis and a in a significant increase in the dermis (CD1a: ~ 1.5-fold, CD207: ~ 1.6-fold) (Fig. 6), thereby confirming the results of the whole slide image analysis (Fig. 5), suggesting a sensitizer-induced migration of our LC-surrogates. Furthermore, mRNA levels for E-cadherin, which is expressed by keratinocytes and required for the selective adhesion of epidermal cells^48^ and presumed to be involved in the localization and mobilization of LCs in the epidermis^29^, were significantly decreased after NiSO_4_ (~ 2.3-fold) and DNCB (~ 2-fold) treatment. While treatment of the FT skin model with NiSO_4_ induced a significant decrease of CD86 (~ 1.9-fold) and a significant increase of CD83 (~ 1.5-fold), mRNA levels in the epidermis, DNCB treatment caused a 1.5-fold decrease of CD86 and a 1.1-fold increase of CD83 mRNA levels in the epidermis. However, treatment with both sensitizers was accompanied by significant decreased mRNA levels of CD83 (~ 1.7-fold) in the dermis. Notably, after treatment with NiSO_4_ as well as with DNCB the mRNA levels for the migration markers CXCR4 (NiSO_4_: ~ 2.4-fold, DNCB: ~ 3.5-fold) and CCR7 (NiSO_4_: ~ 1.8-fold, DNCB: ~ 2-fold) are decreased significantly in the dermal compartment. Furthermore, mRNA levels for IL-8, which is known to play a crucial role in skin sensitization and inflammation^45^, were significantly increased after DNCB treatment in the epidermal (~ 2.1-fold) and dermal (~ 2.6-fold) compartment and after NiSO_4_ treatment in the dermal (~ 1.5-fold), but not in the epidermal compartment (Fig. 6A,C). Treatment of the LC surrogate skin models with sensitizers for 24 h led to a noticeable decrease of mRNA levels for IL-1β (~ 1.5-fold), which is proposed to be one of the first cytokine secreted in response to topical allergens (~ 15 min after exposure)^49^.

**Fig. 7:**
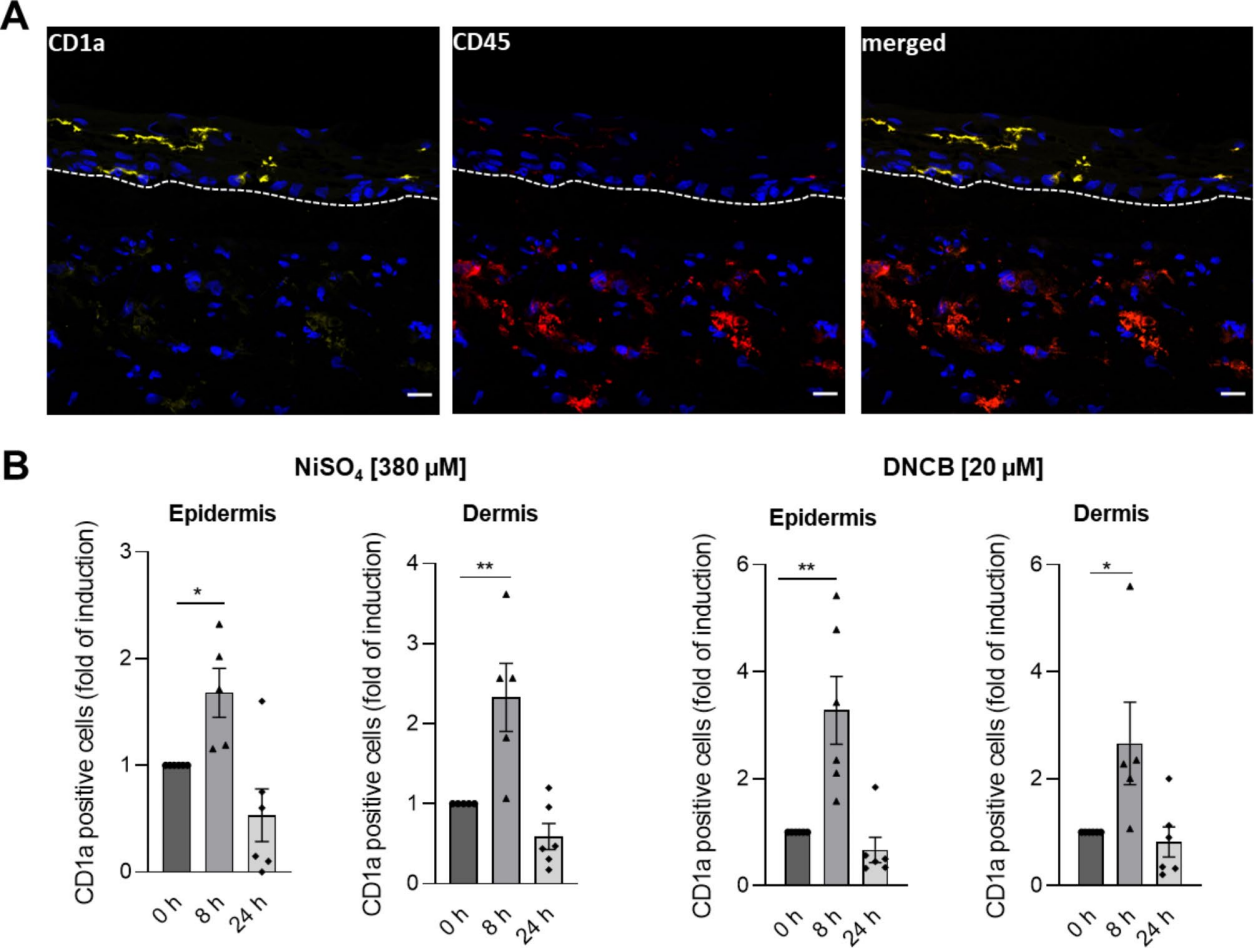
Histological analysis of the full-thickness skin model with incorporated LC surrogates and DDC surrogates. (**A**) Immunofluorescent staining of the immune competent full-thickness skin model including LC and DDC surrogates. LC surrogates were stained with CD1a (yellow signal). DDC surrogates were stained with CD45 (red signal) and Nuclei were stained with DAPI (Blue signal). Scale bar = 20 µm. (**B**) Quantification of the CD1a signal after topical treatment with NiSO_4_ and DNCB for 0h, 8 h, and 24 h was achieved via whole slide image analysis and depicted as fold of induction of CD1a positive cells for the epidermal compartment and the dermal compartment. Error bars indicate the standard errors of the mean (n = 3) independent experiments with each two technical replicates and with *p ≤ 0.05 and **p ≤ 0.01).

Finally, to model immune cell surrogates in the epidermis and the dermis, we incorporated both, LC surrogates and DDC surrogates into the skin. Immunofluorescence staining (Fig. 7A) revealed the successful integration of Mutz-3 derived LC surrogates in the epidermis and the integration of THP-1 derived iDCs as DDC surrogates in the dermis. However, compared to the skin model containing Mutz-LC surrogates only (Fig. 5), sensitizer treatment of the skin models encompassing both, LC and DDC surrogates, did not lead to an increased number of CD1a positive cells in the epidermis or dermis after 24 h of treatment. Remarkably, whole slide image analysis revealed a significant increase of CD1a positive cells in the epidermal and dermal compartment after 8 h of treatment with 380 µM NiSO_4_ (epidermis: ~ 1.7-fold; dermis: ~ 2.3-fold) and 20 µM DNCB (epidermis: ~ 3.3-fold, dermis: ~ 2.7-fold) (Fig. 7B). However, the total number and localization of integrated LCs and therefore the quantity of migrating LCs within two biological replicates as can vary (Fig. 7B). Nevertheless, computing the relative number (fold of induction) of CD1a positive cells in the epidermal and dermal compartment before and after NiSO_4_/DNCB treatment, the results show the same trend indicating a significant sensitizer induced migration of LCs from the epidermis to the dermis, overall. Hence, incorporation of DDC and LC surrogates leads to an early (8 h) sensitizer induced increase of CD1a positive cells in the epidermis and dermis, which, compared to the models with Mutz-LCs only, appears to be diminished after 24 h of treatment.

## Discussion

The aim of this study was to integrate LC surrogates derived from the human myeloid leukemia cell line Mutz-3, and DDC surrogates derived from the human leukemia monocytic cell line THP-1 into a FT skin model. To this date, distinct protocols for the differentiation of Mutz-3 cells and the integration of Mutz-3 derived LC surrogates have been described by various sources^14,15,50^. In line with the published literature, we confirmed the differentiation of Mutz-3 cells into LC surrogates by a high expression (> 85%) for both LC specific markers CD1a and CD207. While most of the published protocols obtain a number of CD1a positive cells between 34 and 89% and of CD207 positive cells between 24 and 73%^14,15,51,52^ after 7–10 days of differentiation using differentiation medium supplemented with 20% fetal bovine serum (FBS)^14–16,51,52^, we were able to decrease the FBS concentration to 5% and yet obtaining a surface marker expression of >85% for CD1a as well as for CD207 after 9 days of differentiation without any medium exchange or additional cytokine supplementation. Thus, our differentiation protocol is favourable in terms of costs regarding the amount of FBS and cytokines used, as well as according to the 3R principle by reducing the FBS concentration considering the animal welfare concerns using FBS.

To validate the functionality in vitro, we exposed the Mutz-LCs to the two commonly used sensitizers NiSO_4_ and DNCB and investigated the changes of the surface marker expression, activation of intracellular inflammatory pathways and expression of inflammatory cytokines. Similar to the results described for CD1a^+^ and CD207^+^ DCs derived from CD34^+^ hematopoietic progenitor cells^38^, but not yet for Mutz-LCs, treatment of our Mutz-LCs with NiSO_4_ and DNCB resulted in an increased MFI for HLA-DR, but decreased MFI for CD1a and CD207. Due to the initially high expression of CD54 and CD86 (~ 99% and ~ 93%), the surface marker expression could only be increased marginally. However, in line with the published literature^53^ a significant increase in the number of cells expressing the maturation marker CD83 was observed after NiSO_4_ (3.0-fold) exposure. Furthermore, we could demonstrate a 1.5-fold induction in the number of CD83 positive cells after DNCB treatment, which was observed in a similar manner for CD1a^+^ and CD207^+^ DCs generated from cord blood derived CD34^+^ cells^38^. Intriguingly, various studies demonstrated for DC surrogates derived from distinct cell types a fundamental role of the inflammatory pathways NF-κB and p38 MAPK in the sensitizer-induced upregulation of CD80, CD86 and CD83, as well as for the secretion of inflammatory cytokines such as IL-1, IL-6, IL-8 or IL12 by DCs^17,38–41^. We were able to prove the NiSO_4_ induced activation of the NF-κB pathway via IκBα-degradation and the phosphorylation of p38 MAPK upon NiSO_4_ as well as DNCB treatment in our Mutz-LCs, suggesting a similar activation manner for LCs as published for DCs, including DDCs, and confirming the expected ability to respond to sensitizers as required for DC activation.

Furthermore, we were interested in the sensitizer-induced responsiveness of Mutz-LCs via inflammatory cytokines. In fact, we could prove significantly increased mRNA levels for IL-6, IL-8 and IL-12p40 and minor increased mRNA levels for IL-1α upon DNCB exposure. Furthermore, treatment with NiSO_4_ resulted in significantly increased mRNA levels for IL-8, IL-1α and IL-1β and minor increased mRNA levels for IL-6 and IL-12p40. Thus, in terms of inflammatory cytokines LCs respond again in a similar manner to NiSO_4_ and DNCB exposure as published for various DC surrogates^17,37,39–41^.

The integration of Mutz-3 derived LC surrogates into tissue-equivalents of the skin has been published previously^11,14–16^. Furthermore, the migration of integrated Mutz-LCs upon NiSO_4_ exposure was demonstrated, but the concentrations chosen for topical treatment with a range from 6.5 to 19 mM are quite high^15,16^ and concentrations of 10–19 mM were required to induce a significant migration of CD1a^+^ cells^15,16^. Contrary, we were able to induce a significant reduction of CD1a^+^ cells in the epidermis and a significant increase in the dermis after topical exposure of only 380 µM NiSO_4_. While the mentioned LC-models were exposed to NiSO_4_ for 16 h^15,16^, our LC-models were treated for 24 h. However, it seems unlikely, that additional 4 h of treatment result in such tremendous differences regarding the LC migration upon sensitizer treatment. Moreover, differences in the treatment concentrations required to induce a significant migration of CD1a^+^ LC surrogates could be caused by various technical and biological aspects, due to the complexity that comes along with engineered tissue comprising multiple distinct cell types. In fact, it needs to be considered that the immune response is not alone mediated by the immune cells, but also by the keratinocytes and fibroblasts, secreting important cytokines and chemokines such thymic stromal lymphopoietin (TLSP) or CXCL12^32,54,55^. Even though keratinocytes (KCs) and fibroblasts (FBs) from neonatal/juvenile foreskin were used for all tissue equivalents^15,16^, donor variations leading to different epidermal thickness, number of epidermal layers or dermo-epidermal adhesion^56^, may impact the immune response. Furthermore, despite the chosen cytokines (GM-CSF, TGF-β and TNF-α) for the differentiation were the same for all LC-models, differences in in the differentiation protocols, including the serum concentration (20%^11,15,16^ vs. 5%), differentiation time (e.g., 7 days^15,16^ vs. 9 days). Furthermore, the seeding protocol for the tissue equivalents varies in the number of integrated LC surrogates (0.5 × 10^6^^15^–1 × 10^6^ cells), the KC:LC ratio (1:2^16^ vs. 1:1^15^) the matrix and collagen source (rat^15,16^ vs. bovine) of the dermis as well as in the media composition (+ serum (substitute)^15,16^ vs. no serum; + hydrocortisone^15,16^ vs. no hydrocortisone) of the ALI-medium. Indeed, crucial differences between the chosen dermis constructs can be identified. While for the published LC-models a simple hydrated collagen was used as matrix for the dermal compartment^11,15,16^, our dermis is characterized by a solid, porous collagen matrix, which allows the fibroblasts to migrate into the scaffold and to synthesize and secrete extracellular matrix components such as elastin and fibrillin-1, mimicking the elastic network of native human skin^57,58^ and thereby potentially providing the required environment for LCs. However, the composition of the ALI medium, in particular the supplementation with hydrocortisone is most likely to be one of the major factors for the reported lower sensitivity and higher treatment concentration up to (10–19 mM^15,16^ vs. 380 µM NiSO_4_) needed for the sensitization and induction of LC migration. In the cell culture hydrocortisone is utilized to support the growth and differentiation of keratinocytes and therefore commonly used for keratinocyte medium and engineering of skin models^58,59^. However, hydrocortisone is a synthetic glucocorticoid, with anti-inflammatory properties frequently prescribed for inflammatory skin diseases such as CHS^60^. Moreover, DCs exposed to glucocorticoids, showed lower expression levels of CD80, CD83 and CD86 and IL-12, resulting in suppressed DC activation and maturation^61,62^.

Since in the past decades, studies investigating DC activation, including LCs and DDCs activation, have been conducted almost exclusively in animal models, mostly in mice, which do not display the human anatomy and LC/DC subsets^9^, we aimed to mimic and monitor the sensitizer-induced LC activation in our engineered immune competent tissue of the skin. Thus, after topical exposure to NiSO_4_ or DNCB, the epidermis and dermis were separated and mRNA levels of LC specific markers, maturation and migration markers were determined. First of all, the migration of LC surrogates induced by the exposure of NiSO_4_ and DNCB could be confirmed by significant lower mRNA levels of CD1a and CD207 in the epidermis and enhanced levels in the dermis. Furthermore, after topical application of both sensitizers, significant lower numbers of E-cadherin could be observed, contributing to the hypothesis that E-cadherin is involved in the retention and migration of LCs^48^. Even though we could confirm the sensitizer-induced LC migration from the epidermis to the dermis on protein and mRNA levels for CD1a, mRNA levels of the migration markers CXCR4 and CCR7 were significantly reduced in the dermal compartment after 24 h of exposure to NiSO_4_ or DNCB. While, Bock et al. could demonstrate a minor increase of CXCR4 mRNA levels in the dermal compartment upon DNCB treatment, they could not observe any significant induction of CXCR4 upon DNCB exposure of skin models with integrated LCs derived from Mutz-3 cells or from PBMCs^11^. However, analysis of the CXCR4 mRNA levels in the regular skin model without immune cells indicate that CXCR4 is highly expressed by keratinocytes and fibroblasts, and epidermal mRNA levels in the skin model with integrated Mutz-LCs are only marginally higher. Hence, the CXR4 expression of keratinocytes and fibroblasts could also be affected, making it difficult to formulate hypotheses that relate to LCs only. Notably, in line with the observed sensitizer-induced secretion of IL-8 by isolated LC surrogates^50^, we could determine a significant increase of IL-8 mRNA levels in the dermal compartment upon NiSO_4_ and DNCB exposure. In conclusion, we could elucidate that sensitization of Mutz-LCs is accompanied by similar molecular events observed in DC activation, including the activation of p38 MAPK and NF-κB as well as the increase in mRNA levels of IL-6, IL-8, IL-1 and IL-12p40. Furthermore, we could prove that our Mutz-3 derived LC surrogates can be integrated as functional LC surrogates, displaying the sensitizer-induced molecular events of maturation and migration not only in 2D, but also in a 3D tissue equivalent. Hence, our model can be used to study the molecular events of LC activation and maturation in vitro and potentially for sensitizer identification and drug discovery.

In a final step, we were able to integrate functional LC surrogates and DDC surrogates into the FT skin model. However, compared to the Mutz-LC model, exposure of the of the skin models comprising LC and DDC surrogates to NiSO_4_ or DNCB for 24 h, did not lead to an increased number of CD1a positive cells in the epidermis or dermis. Remarkably, after 8 h of treatment with NiSO_4_ or DNCB, a significant increase of CD1a positive cells in the epidermal and dermal compartment could be observed. One vague hypothesis could be that due to the presence of DDCs, which have been proposed to migrate faster and in a larger number than LCs to the lymph nodes^7^, some sort of cross-talk and transfer of the captured antigens by LCs to DDCs might occur, followed by LC apoptosis. In fact, Langerin mediated LC-DDC cross-talk and antigen transfer of HIV-1 has been reported^63^. In addition, it was proposed that LCs might transfer targeted antigens to DDCs^64^. In line with our results, in vivo sensitization studies conducted in guinea pigs, revealed an increase of LCs in the epidermis and dermis within 4–6 h after DNCB exposure, but first ultrastructural signs of cell damage in LCs 6–12 h after exposure and between 19 and 24 h a notably decrease in LC numbers^65^. Furthermore, exposure of human skin with nonanoic acid led to a significant decrease in Langerhans cells and induction of apoptosis after 24 h of treatment^66^. However, the precise molecular events, including the potential cross-talk between LCs and DDCs remains elusive.

## Electronic supplementary material

Below is the link to the electronic supplementary material.

Supplementary Material 1

## Data Availability

Data is provided within the manuscript or supplementary information files.
