# Supplementary material for: Incorporating immune cell surrogates into a full-thickness tissue equivalent of human skin to characterize dendritic cell activation
Source: Sci Rep. 2024 Dec 4;14:30158. doi: 10.1038/s41598-024-81014-9 (PMC11615323; doi:10.1038/s41598-024-81014-9)
Supplement: Supplementary file 1 — Supplementary Material 1 [file 41598_2024_81014_MOESM1_ESM.docx]

**Supplementary Information**


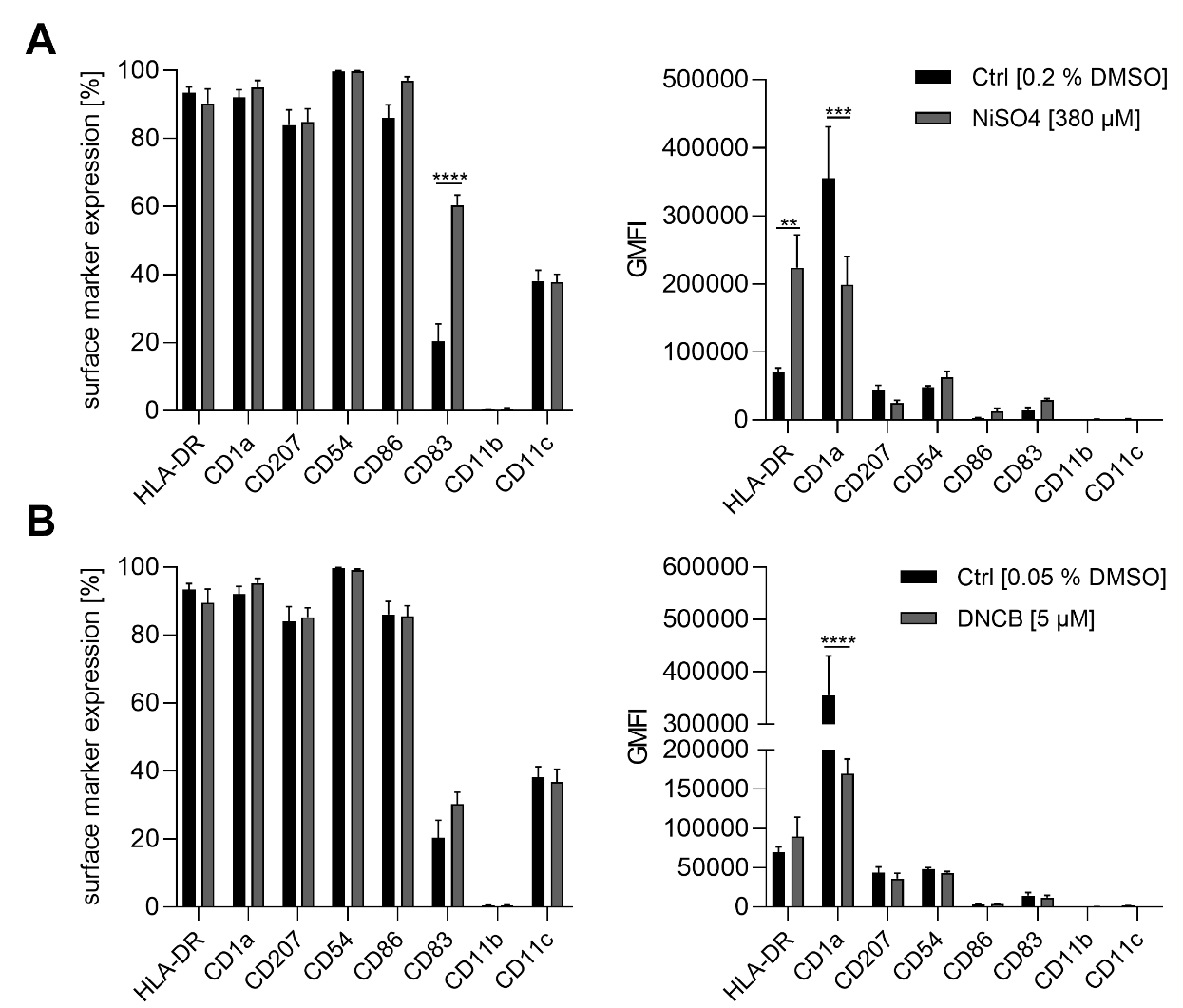


**Supplementary Figure 1** Surface marker expression of Mutz-3 derived LCs after sensitization, depicted as percentage of positive cells and as geometric mean fluorescence intensity (GMFI). 2 × 10^5^ Mutz-LCs/ mL were seeded in 4 mL MEM α supplemented with 5% FBS, 1% P/S and 0.05 mM 2-mercaptoethanol into a 12-well plate and exposed to **(A)** NiSO4 [380 µM] or **(B)** DNCB [20 µM] for 24 h. Surface marker expression of at least 10,000 viable cells was analyzed via flow cytometry. Error bars indicate the standard errors of the mean (n = 3 independent experiments with ** = p ≤ 0.01, *** = p ≤ 0.001, and **** = p ≤ 0.0001).


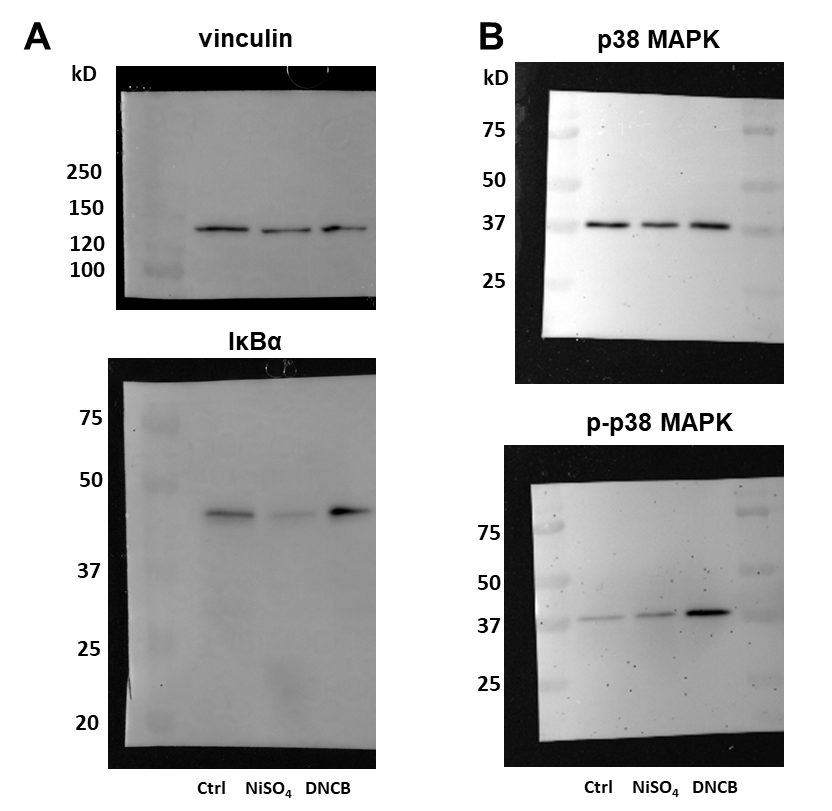


**Supplementary Figure 2** Full length western blots according to Figure 2. **(A)** Degradation of IκBα after NiSO_4_ [500 µM] and DNCB [25 µM] treatment for 1 h. **(B)** phosphorylation of p38 MAPK after NiSO_4_ [500 µM] and DNCB [25 µM] treatment for 30 min. Depicted is one representative blot of three independent experiments.
